# Dynamic Evolution of Antimicrobial Peptides Underscores Trade-Offs Between Immunity and Ecological Fitness

**DOI:** 10.3389/fimmu.2019.02620

**Published:** 2019-11-08

**Authors:** Mark A. Hanson, Bruno Lemaitre, Robert L. Unckless

**Affiliations:** ^1^School of Life Science, Global Health Institute, École Polytechnique Fédérale de Lausanne, Lausanne, Switzerland; ^2^Department of Molecular Biosciences, University of Kansas, Lawrence, KS, United States

**Keywords:** innate immunity, antimicrobial peptide (AMP), molecular evolution, population genetics, diptericin, drosophila, diptera, attacin

## Abstract

There is a developing interest in how immune genes may function in other physiological roles, and how traditionally non-immune peptides may, in fact, be active in immune contexts. In the absence of infection, the induction of the immune response is costly, and there are well-characterized trade-offs between immune defense and fitness. The agents behind these fitness costs are less understood. Here we implicate antimicrobial peptides (AMPs) as particularly costly effectors of immunity using an evolutionary framework. We describe the independent loss of AMPs in multiple lineages of Diptera (true flies), tying these observations back to life history. We then focus on the intriguing case of the glycine-rich AMP, *Diptericin*, and find several instances of loss, pseudogenization, and segregating null alleles. We suggest that *Diptericin* may be a particularly toxic component of the Dipteran immune response lost in flies either with reduced pathogen pressure or other environmental factors. As *Diptericins* have recently been described to have neurological roles, these findings parallel a developing interest in AMPs as potentially harmful neuropeptides, and AMPs in other roles beyond immunity.

## Introduction

The innate immune system plays a vital role in host defense against pathogens. This is particularly true in invertebrates, which lack an adaptive immune system. Antimicrobial peptides (AMPs) are one of the main effector molecules of innate immunity in many organisms and, as such, they represent the front lines in the coevolutionary struggle between host and pathogen. AMPs are often cationic, amphipathic peptides that defend their hosts against infection by disrupting the cell membranes of invading microbes (1). However, the dose makes the poison, and AMPs can also be toxic to eukaryotic host cells under certain conditions. This suggests that host immunity needs to strike a delicate balance: AMPs need to be potent enough to quickly inhibit pathogenic microbes, but not so potent that they upset the balance of the microbiota or damage host tissue.

Indeed, many pathologies in humans have been observed when this balance is perturbed. These include chronic inflammatory skin or bowel diseases (2–4), and pulmonary infections including cystic fibrosis wherein reduced levels of β-Defensins and the cathelicidin LL-37 are associated with increased risk of infection (5–9). The cathelicidin LL-37 is implicated in autoimmune reactions because it can be toxic to white blood cells (10), induce inflammation in the nervous system (11), or even damage host tissues during anti-cancer responses (12). Recent studies have also suggested the Alzheimer's peptide Amyloid-beta is an AMP in the nervous system, and that Alzheimer's may in part be an infectious disease (13–15). These observations of AMPs as toxic agents are further supported by reduced lifespan in *Drosophila* fruit flies ubiquitously expressing AMPs in the brain (16), or systemically (17). During aging, *Drosophila* NF-κB signaling is also implicated in neurodegeneration with AMPs as prime suspects (18). Thus, AMP dysregulation can impose a significant threat to organismal health.

Insects, and particularly *Drosophila melanogaster*, have been integral to unraveling the innate immune response, including the regulation of AMPs by the Toll and Imd NF-κB signaling pathways (19). Thus, far seven AMP gene families have been described in *Drosophila*: *Defensin, Cecropin, Attacin, Diptericin, Drosocin, Metchnikowin*, and *Drosomycin*. Another class of AMP-like effectors called the *Bomanins* are also essential for Toll-mediated defense, however their antimicrobial properties await functional clarification (20, 21). *Drosophila* AMP evolution is shaped both by balancing and diversifying selection at the sequence level (22, 23). Following a duplication event and subsequent speciation, *Drosophila Diptericins* rapidly diverged into distinct *Diptericin* clades (24). In contrast, balancing selection seems to maintain a stable polymorphism amongst alleles that provide either moderate or poor protection against systemic infection with *Providencia rettgeri (P. rettgeri)* (25). Why selection should favor *Diptericin* alleles that result in loss of immune competence is unclear. One possibility is that the immune-poor *Diptericin* allele is selected for through trade-offs between poor immune defense when infected and higher fitness when uninfected. Indeed, rare *Diptericin* null alleles are observed in North American populations (25), and patterns of duplication and loss in *Diptericin* and other *Drosophila* AMPs have resulted in copy number variations amongst species (24, 26–28).

As AMP dysregulation can affect health, copy number variation may impose a significant challenge for the maintenance of optimal gene expression (29). Yet perhaps the most overt patterns in AMP evolution are duplication events affecting copy number, which is widespread in both humans and fruit flies (30–34). Therefore, conflict between maintenance of healthy expression levels and improved immune competence may drive patterns of AMP gain/loss or changes in expression patterns. In this model, we expect that AMPs are evolutionary liabilities in the absence of infection, and that host ecology and associated pathogen pressure will drive the evolution of AMP content both at the level of broad AMP gain/loss, and also of AMP expression: species with strong pathogen pressures would evolve to increase potential AMP production, while species whose ecologies involve less exposure to pathogens would be expected to reduce their AMP complement.

While characterizing pathogen pressure in different animal hosts is exceedingly difficult, we can use host ecology as a proxy for infectious pressures. The use of sterile food resources (such as plant sap) reduces the opportunities for microbes to inoculate hosts. There are several insects that spend large portions of their lives (larval, adult, or both) feeding on sterile or near sterile food resources—likely reducing the evolutionary benefits of AMPs and/or AMP induction. The pea aphid (which feeds on sterile plant phloem) is one such insect that has lost not just effectors, but also an entire NF-κB immune signaling pathway (35). Loss of immune signaling is also observed in plant-feeding *Tetranychus* mites (36, 37), as well as bed bugs (38) and body lice (39), suggesting blood-feeding may be a similarly clean feeding ecology. It should be noted that in some cases these hosts have intimate associations with endosymbionts, microbes that supplement nutrition or protect against infection. One argument to explain loss of immune signaling is that it is a direct consequence of endosymbiont presence to avoid negative consequences associated with chronic activation of the host immune response (40). However, cereal weevils live in sterile environments and have nutritional endosymbionts, but they instead utilize AMPs to regulate their symbiont populations (41). Thus, what factors of sterile lifestyles and/or endosymbionts promote immune gene loss remains poorly resolved.

As AMP copy number evolves rapidly, we suspected AMP evolution might respond to shifts in host ecology before entire immune pathways are affected. To test this, we surveyed Diptera (true flies) for AMP presence or absence and interpret this in the context of host ecology. Diptera are an extremely diverse lineage with equally diverse and unique ecologies and life histories, and boast numerous sequenced genomes and transcriptomes. We probed these diverse flies for classic AMP families described in *Drosophila* and other insects to better understand the forces driving AMP gain or loss. We further analyzed *Drosophila* copy number and sequence variation in conserved AMPs, tying these results back to life history. Globally, we describe a pattern suggesting AMPs are lost in Diptera lineages with more sterile life histories, with striking parallels to loss of immune signaling in other arthropods with sterile food resources. We also focus on *Diptericin*, which we suggest is a particularly costly AMP, describing distinct evolutionary patterns across ecologically diverse *Drosophila* and within *D. melanogaster*.

## Results

### Some AMP Families Are Absent in Diptera Living in More Sterile Environments

**Figure 1:**
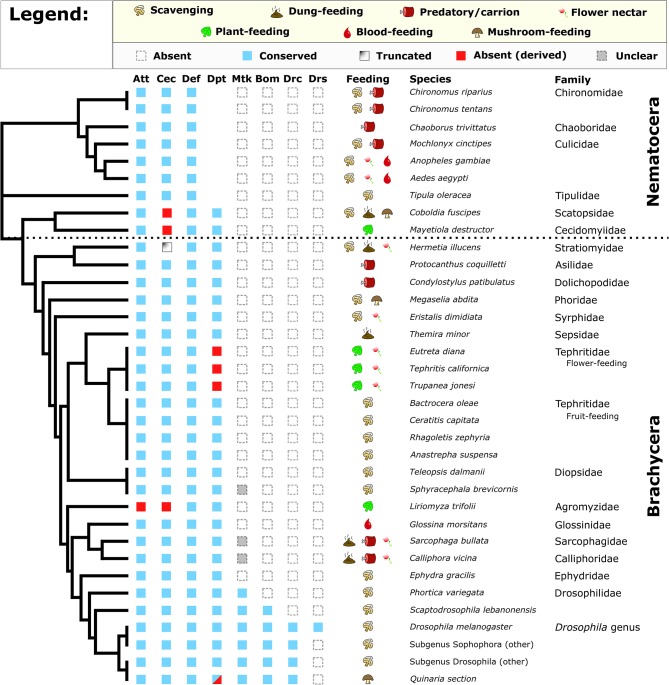
Conservation of *Drosophila* AMP families in diverse Diptera. Broadly, *Attacin, Cecropin, Defensin, and Diptericin* are conserved in most flies. However, *Cecropins* are absent or truncated in select lineages, while flower-feeding Tephritids and *Liriomyza trifolii* lack subsets of AMP*s*. We also recover a pattern of *Diptericin* loss in members of the Quinaria section of *Drosophila* (shown later). Full annotation of larval and adult feeding ecologies is given in Supplementary Data File 1. Cladogram adapted from Vicoso and Bachtrog (43).

Diptera diverged from other related holometabolous insects about 272 mya [timetree.org; Kumar et al. (42)] and diversified into extremely broad ecological habitats. We surveyed 31 Dipteran genomes as well as diverse *Drosophila* species for the presence of eight AMP/AMP-like families either described in *Drosophila* (*Bomanins, Drosocin, Drosomycin, Metchnikowin*) or characterized more broadly across Dipterans and other insects (*Diptericins, Cecropins, Attacins, Defensins*). We also annotated the feeding ecologies of these diverse flies to better understand which lineages may have reduced pathogen pressure owing to food resource use (Supplementary Data File 1). We performed an iterative reciprocal BLAST search using known AMPs against genomic or transcriptomic sequence. We found that *Drosocin, Metchnikowin, Bomanins*, and *Drosomycin* are restricted to *Drosophila* and their close relatives (Figure 1). Using a lenient E-value threshold, we were able to recover *Metchnikowin* from diverse mushroom-feeding *Drosophila* and perhaps other flies, and confirmed their identities by alignment and reciprocal BLAST (Figure S1), improving on previous annotations of immune genes in this lineage (24). The other AMP families show a broader taxonomic distribution (Figure 1).

One striking pattern is the absence of *Cecropin* in two Nematocerans: the plant-feeding Hessian fly *Mayetiola destructor* and the oyster mushroom pest *Coboldia fuscipes*. The *Coboldia* genome is a small, well-assembled genome (~100 Mbp, scaffold N50 = 242 Kbp) (43, 44), and *Cecropins* throughout Diptera share similar motifs from the N terminus to C terminus. As such, we interpret this absence of *Cecropin* in both *M. destructor* (plant sap-feeding) and *C. fuscipes* (scavenger-feeding) as a likely true loss of *Cecropin* in this basal lineage. We also found a partial *Cecropin* sequence truncated by a premature stop codon in *Hermetia illucens* (scavenger-feeding). Finally, we did not recover *Cecropin* from the genome of the leafminer *Liriomyza trifolii*, an independent transcriptome of *L. trifolii* pupae (a life stage when AMPs are often highly upregulated), or from a sequence read archive (SRA) file (GenBank accession: DRX064600) of the related *Liriomyza chinensis*. We see no immediate pattern in feeding ecology or life history that predicts *Cecropin* loss, but we also failed to recover an *Attacin* from *Liriomyza*, suggesting *Liriomyza* has lost AMPs from two gene families (*Cecropins* and *Attacins*). Called “leafminers,” *Liriomyza* larvae feed internally in the leaves of plants, an environment protected from external microbes by the immune system of the host plant; a more sterile food resource than most Dipterans. Moreover, we also failed to recover *Diptericin* in three flower-feeding Tephritid species. Like the leafminers, these flower-feeding flies live in a protected environment owing to larval development inside budding flower inflorescences (45).

Within the genus *Drosophila*, we observed two unique instances of AMP gain/loss we note separately. First, the genome of the cosmopolitan fly *Drosophila busckii* encodes no less than nine intact *Diptericin* genes, and we further recovered three pseudogenes in the *D. busckii Diptericin* gene region (Figure S2); for context, other *Drosophila* typically have only 2–3 *Diptericin* genes (24). *Drosophila busckii* is a cosmopolitan generalist species in common association with *D. melanogaster*, however *D. busckii* arrives later to rotting fruits and compost relative to *D. melanogaster* (46, 47). To favor the retention of so many *Diptericin* copies suggests the *Diptericin* response is highly important *for D. busckii* ecology. Second, we found that one paralog of the *Attacin A/B* duplication event has been lost in *Drosophila sechellia*, a species closely-related to *D. melanogaster*. *Drosophila sechellia* is famous for its unique ecology, feeding on toxic morinda fruit that repels other flies (48). Beyond losing this *Attacin* paralog*, D. sechellia* also lacks the ability to encapsulate and kill invading parasitoid wasps, associated with loss of function in immune genes involved in the melanization and stress responses (49). It seems likely that the toxins in morinda fruit would repel parasites such as wasps, reducing infectious pressure on *D. sechellia*. Thus, this ecology—already associated with loss of immune genes—may have additionally promoted loss of an *Attacin* as well.

Overall, we observe numerous instances of AMP loss across the Diptera phylogeny. The loss of Cecropins in ecologically diverse lineages is puzzling. For the mushroom pest *C. fuscipes* and Hessian fly *M. destructor*, either scavenging (*C. fuscipes*) or sap-feeding (*M. destructor*) could reflect an ancestral ecology promoting *Cecropin* loss. These two last shared a common ancestor ~250 mya [Timetree; (42)], and transitions from generalist to specialist ecologies, and back again, have been inferred in *Drosophila* (50). However, more strikingly we observe AMP gene family loss in all three strictly plant-feeding fly lineages assessed (the Hessian fly *M. destructor, Liriomyza* leafminers, and flower-feeding Tephritids), reminiscent of immune gene loss in sap-feeding Pea Aphids.

### Parallel Loss of Diptericins in Lineages With Divergent Ecology

**Figure 2:**
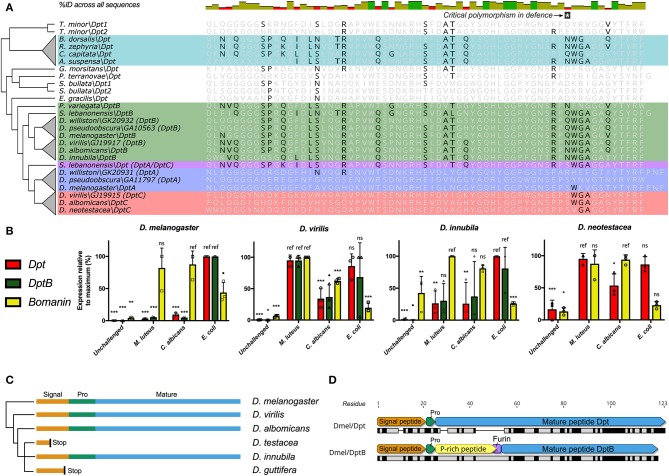
Diptericin evolution in Tephritids and Drosophilids. **(A)** The Diptericin C-terminal sequence of fruit-feeding Tephritids has converged on *DptB*-like residues (also see Figure S3). Major *Diptericin* clades: Blue, *DptA*; Green, *DptB*; Red, *DptC*; Teal, Tephritid *Dpts*, and *S. lebanonensis Dpt* in purple is a direct outgroup to the *DptA* and *DptC* clades (collectively referred to as “*Dpt*” in **B**). The polymorphism critical for defense (25) is indicated, and Tephritid *Diptericins* have converged on the Q/N polymorphism found in the *Drosophila DptB* clade. **(B)** *Diptericin* expression in select lineages of *Drosophila*. Expression is normalized to the maximum for each gene within each species, and statistical comparisons are done with reference to the treatment that induced expression most (see Materials and Methods). **(C)** *DptB* has been pseudogenized in two independent lineages of mushroom-feeding *Drosophila*: *D. testacea* and *D. guttifera*. Signal, Signal peptide; Pro, propeptide; Mature, Mature peptide. **(D)** The mature peptides of *D. melanogaster* Dpt and DptB differ markedly in mature structure, including a critical furin cleavage site in DptB that likely leads to two mature peptides. At the protein sequence level Dpt has only 41.7% pairwise identity to DptB (black bars = identical sites).

In our screen of AMP conservation in Diptera, we were intrigued by the loss of *Diptericin* in some Tephritid fruit flies and some *Drosophila*; *Diptericin* was previously shown to have rare null alleles segregating in a North American *D. melanogaster* population (25). While assembly quality was variable amongst the Tephritid genomes, the absence of *Diptericin* in three independent flower-feeding Tephritid species, but presence in all screened fruit-feeding Tephritid species suggests *Diptericin* is lost in the flower-feeding Tephritid lineage. *Diptericin* is an AMP that has attracted a great deal of attention as the canonical readout of Imd signaling in *D. melanogaster* (19), and for its highly specific interaction with *Providencia rettgeri* bacteria (25, 51). Interestingly, the *Diptericin* sequence retained in the fruit-feeding Tephritids has converged on a Drosophilid *DptB*-like sequence (Figure 2A). Furthermore, it was previously reported that *DptB* was pseudogenized in the mushroom-feeding *Drosophila* species *D. guttifera* and likely also in *Drosophila neotestacea (D. neotestacea)* (24). However, when we screened the recently-sequenced mushroom-feeding *Drosophila innubila* genome, we recovered intact coding sequence for *DptB*. It is possible that the intact *DptB* sequence in *D. innubila* could reflect that *DptB* in mushroom-feeding flies was initially pseudogenized not due to loss of coding sequence, but rather due to mutations affecting gene expression. We therefore performed qPCR following infection to determine the expression profile of *Diptericins* amongst mushroom-feeding flies and included outgroup *Drosophila* to inform our interpretations.

We used a Bomanin (*Bom791*) as a positive control for infections more specific to the Toll pathway in *D. melanogaster*. We further intended to use *Dpt* as a specific readout of Imd signaling, and as an independent control gene for assessing *DptB* expression. First, we found that *DptB* is strongly induced in *D. innubila*, suggesting it is not pseudogenized as in sister lineages. However, we found that *Dpt* expression is highly variable across *Drosophila* species (Figure 2B). *Dpt* is more specifically induced by Gram-negative bacterial challenge in *D. melanogaster*, and indeed we see this pattern in the outgroup flies *Drosophila pseudoobscura (D. pseudoobscura)* and *Drosophila immigrans (D. immigrans)* (Supplementary Data File 1), and also broadly in *D. innubila*. However, *Dpt* and *DptB* are similarly induced by either Gram-negative or Gram-positive bacterial challenge in *D. virilis*, and the same is true for *Dpt* in both *Drosophila testacea (D. testacea)* (not shown), and *D. neotestacea* (Figure 2B, and see Materials and methods). Using Sanger sequencing, we additionally confirmed that *DptB* is pseudogenized in *D. testacea* by a premature stop codon, supporting its absence in the *D. neotestacea* transcriptome (GenBank accession: MN311476). The mutation affecting *DptB* in the Testacea group is distinct from the mutation in the Quinaria group species *D. guttifera* (Figure 2C), suggesting independent loss events.

Thus, the pseudogenization of *DptB-*like genes in both flower-feeding Tephritids and two lineages of mushroom-feeding flies reflects that first there was convergent evolution toward *DptB*-like sequence in both Tephritids and Drosophilids. Thereafter, subsequent independent losses of *DptB*-like *Diptericins* occurred in lineages with ecologies that diverged from fruit-feeding. This pattern suggests *DptB* may be attuned to fruit-feeding ecology, but not as useful in other ecological niches.

We can only speculate on how evolution shapes patterns of *DptB* gain/loss: in *D. melanogaster*, functional characterization of *DptB* was long-ignored in favor of its more potently-induced paralog *Dpt*. However, recent studies have revealed that the two *Diptericins* have markedly different activities in immunity and host physiology. First, Unckless et al. (25) showed that a specific serine allele in *Dpt* confers defense against *P. rettgeri*, however no *DptB* gene in any fly encodes a serine at this site. Alternately, Barajas-Azpeleta et al. (52) found that *DptB*, but not *Dpt*, is required for long-term memory formation. There are a number of overt structure and sequence differences amongst *Dpt* and *DptB* (Figure 2D). First, *Dpt* encodes an 83-residue mature peptide with a proline-rich domain tailed by a glycine-rich Attacin-family domain. This 83-residue mature peptide is secreted following cleavage of the Dpt signal peptide and propeptide. On the other hand, *DptB* encodes a furin cleavage site (RVRR) between its proline-rich and glycine-rich domains, similar to other AMPs of the Attacin gene family (53); In Attacin C, this furin cleavage results in two secreted peptides, a proline-rich AMP (called MPAC) and a separate glycine-rich AMP (54). Furthermore, amongst the many sequence differences between *Dpt* and *DptB* (see Figure 2A) is the aforementioned serine residue of Dpt that confers defense against *P. rettgeri* bacteria. In *Dpt* genes, this residue is polymorphic (S/R/Q/N) in *D. melanogaster* and close relatives (25). However, DptB encodes a polymorphism for only Q/N at this site, including in convergent *DptB*-like *Diptericins* of Tephritid fruit flies. Globally, in *D. melanogaster, Dpt* appears to be key in mediating defense against *P. rettgeri* bacteria, while *DptB* is uniquely required for memory formation. Accordingly, these two *Diptericins* have overt differences in mature peptide products. Here we implicate host ecology as a likely determinant of *Diptericin* evolution, and suggest that these overt differences may have ecology-dependent effects on fitness leading to distinct patterns of gain/loss.

### Null Alleles of Diptericin Are Segregating in Wild Populations of *D. melanogaster*

**Figure 3:**
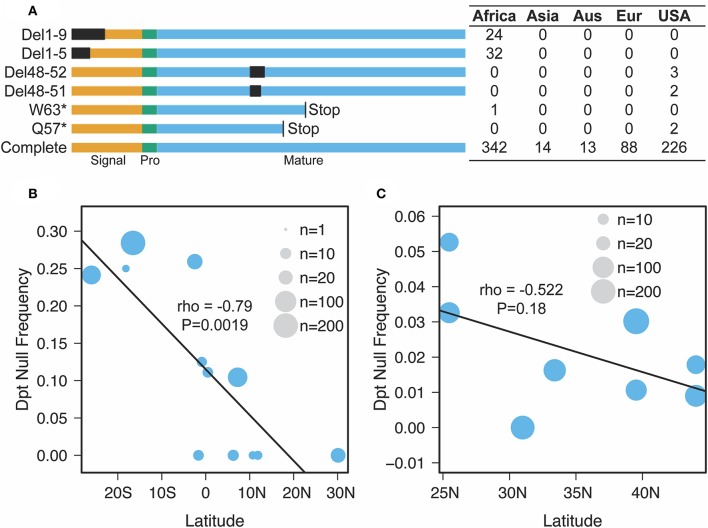
Nature and geographic distribution of *Dpt* null alleles. **(A)** At least six unique null alleles segregate in *D. melanogaster Dpt* worldwide. Del1-9 and Del1-5 are deletions removing the first 9 or 5 amino acids, Del 48-52 and Del 48-51 are in frame deletions that remove amino acids in the mature peptide and W63* and Q57* are premature stop codons. Signal, Pro and Mature correspond to the signal peptide, propeptide and mature peptide of the protein. The box to the right denotes the counts of each allele in each population (Aus, Australia; Eur, Europe, USA, DGRP population only). **(B,C)** The correlation between latitude and null frequency in African **(B)** and North American **(C)** populations (data from pooled sequencing of populations along a cline). The size of the circle represents the number of individuals sequenced **(B)** or reads mapped **(C)** in each population.

Our findings on *Diptericin* evolution coupled with recent descriptions of distinct *Diptericin* activities uniquely position this AMP family for providing insight into how conflicting roles in immunity and physiology can shape AMP evolution. Unckless et al. (25) reported the maintenance of two alternate alleles (serine/arginine) at residue 92 of the full length Dpt protein (residue 69 of the mature peptide) in wild populations of *D. melanogaster* and *D. simulans*. *Providencia rettgeri* is a natural pathogen of *D. melanogaster* (55), yet the *Dpt* arginine allele is maintained by balancing selection in the wild despite being associated with poor immune defense against *P. rettgeri* infection. Additionally, Unckless et al. (25) reported a rare null mutation in a *D. melanogaster* North American population (DGRP) (56) resulting in a premature stop codon affecting ~1% of the reference strains. Surprisingly, when we investigated a set of African populations (DPGP) (57), we found multiple independent null mutations segregating in different sub-populations throughout the Africa sampling range (Figure 3A). Even more surprising, the prevalence of these null mutations reaches over 20% in some populations and appears to follow a latitudinal cline (Figure 3B). As such, selective pressure on *Dpt* may follow a clinal gradient in Africa, favoring *Dpt* loss in southern African populations. Note that the cline crosses the equator and so may not be driven by climate alone. We also recover a similar, though not significant, trend for null alleles segregating in North American collections (Figure 3C).

These data suggest that despite the described importance of *Dpt* in defense against the ecologically relevant pathogen *P. rettgeri*, null alleles associated with extremely poor immune defense are actively segregating in wild *D. melanogaster*. This suggests that the evolutionary forces behind *Diptericin* loss are not entirely passive. Taken together with the loss of *DptB* in other flies, instead this implicates active selection on *Diptericins* as peptides deleterious for fitness in alternate ecological conditions.

## Discussion

AMPs must maintain a fine balance: being potent enough that they can kill harmful pathogens but not so harmful that they damage host tissues directly or by damaging beneficial components of the host's microbiome. It stands to reason that host ecology drives pathogen pressure and therefore might indirectly shape the complement of AMPs in a given host. In our survey of AMPs across Diptera and within *Drosophila* presented above, we find some support for an adaptive loss of AMPs in hosts associated with more sterile habitats. There is increasing awareness that these classic immune molecules can play diverse physiological roles, and that evolution may be shaping AMP copy number and sequence due to selection on non-immune functions. When considering the internal plant parasites of the Tephritid family and the leaf miner, clear parallels can be drawn regarding sterile food resource use and other fluid-feeding arthropods that have similarly lost or re-organized their immunity genes, namely: aphids, some mites, bed bugs, and body lice (35–39). It may also be that these plant-parasitic flies have yet-uncharacterized bacterial endosymbionts that impose selection against certain AMPs, enabling their specialist lifestyle. We also describe multiple incidents of *Diptericin* loss in *Drosophila*: *DptB* in some mushroom-feeding flies, a lineage with a specialist ecology whose microbiota differs drastically from *D. melanogaster* (58), while *Dpt* null alleles are segregating in wild *D. melanogaster* populations.

The central question then becomes: why should immune-inducible AMPs, antimicrobial agents required for competent host defense, be lost so readily? We can think of two evolutionary scenarios that would lead to the loss of AMPs. First, when infection pressure is low, relaxed constraint on protein sequence and/or expression could lead to the accumulation of mutations that compromise protein function and eventually lead to pseudogenization and loss. This represents a neutral process where genetic drift is the force removing AMPs. The second evolutionary scenario is that AMPs are costly in the absence of infection, so when infection pressure is low, mutations that compromise function (indels, premature stop codons, cis-regulatory mutations) are actually selected for. If periods of low infection pressure persist long enough, those mutations can become fixed, and gene function is lost.

Several lines of evidence support the second, “selective loss” scenario. First, if relaxed constraint in the absence of infection drove AMP loss, we would expect the loss of AMPs to be somewhat random. However, we see convergent loss of *DptB-like* genes in independent lineages with divergent ecologies, and *Dpt* null alleles segregating in wild populations of *D. melanogaster*. Of course, if AMPs are specific to a small suite of pathogens (e.g., *Dpt* and *P. rettgeri*), perhaps those pathogens are relatively absent in some natural populations compared to others. This would also lead to increased loss of AMPs, but *via* neutral processes. In the case of *Dpt*, however, *Providencia rettgeri* is distributed worldwide including throughout Africa (59, 60). Therefore, it is unlikely to be completely absent from African populations of *D. melanogaster* where null alleles are common. Instead, the specific loss of *Diptericins* in *Drosophila* might reflect a deleterious consequence when dysregulated in non-immune tissues. For example, AMP expression increases dramatically in the head tissue of aging flies (18). This explanation seems more likely, as non-cell autonomous *DptB* is known to affect memory formation in *D. melanogaster* (52), evidence of direct *Diptericin* impact on brain function. A second line of support for the “selective loss” scenario is the null allele cline observed in African populations alongside a parallel (though not significant) cline in North America. Such parallel clines are often used as evidence for selection acting on alleles (61–63). While neutral processes could lead to clines in null alleles as well, with the null allele spreading from an initial source population, the likelihood of this happening in parallel on two continents is small. Finally, there is growing evidence that several AMPs may inflict damage on host tissues. For instance, the cathelicidin LL-37 is toxic to leukocytes (64), and constitutive expression of AMPs reduces lifespan in *Drosophila* (17). These direct observations of deleterious effects strongly undermine the idea that neutral processes are driving the loss of AMPs, instead suggesting these molecules impose a significant effect on host fitness.

One exciting explanation for AMP fitness costs is the idea that AMPs are dysregulated through aging, leading to chronic inflammatory responses and eventually cell death. Additionally, the idea that AMPs may be active in the nervous system is an attractive recent hypothesis that demands more consideration (65, 66), particularly to understand the roles these short peptides play in neuronal homeostasis (67). For instance, while implicated in Alzheimer's disease for decades, the specific nature of how Amyloid-beta contributes to dementia remains unclear (68). Understanding its role in immunity may shed light on the cause and progression of amyloid plaques (13), and reveal the true culprit(s) behind Alzheimer's progression; an interesting recent study found that Amyloid-beta binds to the human cathelicidin LL-37, forming heterodimers that reduce the toxicity of LL-37 to host cells (69). Alongside evidence that Alzheimer's may be an infectious disease (14), dysregulation of AMPs in the nervous system upon chronic infection could lead to host cell toxicity. Appreciating the role of AMPs in the nervous system, particularly during infection, may lead to breakthroughs in treating neurodegenerative disorders such as Alzheimer's disease or Parkinson's disease.

If indeed AMPs are deleterious in non-immune contexts, this may promote balancing selection in populations with dynamic immune pressures. Beyond AMPs, trade-offs between immunity and fitness are well-documented, implying that an immune system is advantageous only in the context of immune challenge, but otherwise is detrimental to reproductive fitness (70–73). As the front line of innate immunity, AMPs should be primary actors on this evolutionary stage, and selection for or against immunity genes should therefore act strongly on context-dependent AMPs. Recent studies report that both balancing and diversifying selection has shaped the *Drosophila* AMP arsenal (22–25), revising how we view AMPs as actors in host-pathogen interactions (28). If balancing selection is driven by trade-offs between alleles that provide increased resistance during infection but are costly when hosts are uninfected, this could explain the dynamic patterns of AMP gain and loss described here. By characterizing e.g. *Diptericin* loss throughout Diptera, we provide the beginnings of an immunological fossil record with extinct (pseudogenes) and extant *Diptericin* gene copies in different lineages. The observations of other AMP gene losses throughout Diptera extend this fossil record back in time, describing lineages with different stages of loss stemming from an ancestral immune-competent fly to derived lineages lacking subsets of certain AMPs.

Globally, we highlight how host ecology predicts AMP loss, and follow the evolution of AMP lineages throughout Diptera. We describe that selection on the innate immune system can act swiftly and directly on AMPs, implicating some AMPs as deleterious molecules in the absence of microbial challenges. These results could relate to the newly discovered role of AMP-like peptides in neurodegenerative diseases and autoimmune disorders. If so, our findings offer evolutionary signatures supporting the notion that trade-offs between immunity and fitness are mediated by costs related to the maintenance of autotoxic host AMPs.

## Materials and Methods

### Survey of AMP Families in Published Diptera Genomes and Transcriptomes

We first conducted a thorough literature review to annotate the life histories of diverse Diptera. We then searched for *Drosophila* AMP families present in other Diptera using an iterative step-wise tBLASTn approach followed by manual curation; of note, we used an extremely lenient E-value for the shortest peptides (e.g., E <100 for Mtk, 26 residues long), followed by manual curation. In brief, we collected AMP genes from sequenced *Drosophila* and then BLASTed all available orthologs against outgroup genomes from Vicoso and Bachtrog (43). We collected all confirmed outgroup orthologs and re-performed this BLAST against any species where no match was found, until we ceased to recover new orthologs. To verify any patterns of loss we observed (e.g., *Dpt* loss in Tephritid species), we further searched for outgroup sequence data (genomes, transcriptomes, or raw SRAs) to include in our analyses as independent databases. For some orthologs, only a partial sequence was recovered on a scaffold assembled with many gaps (NNNs). If sequence similarity was highly conserved we annotated these AMPs as “present” but do not include them in phylogenetic analyses as their information content was poor. All sequence databases used in this study are included in Supplementary Data File 1. Sanger sequencing results are deposited in GenBank under accessions: MN311474-MN311476.

To investigate sequence similarity and validate curated orthologs, we aligned sequences using MAFFT and performed phylogenetic analysis using Neighbor-joining (1000 bootstraps) and/or Maximum likelihood (100 or 500 bootstraps) methods implemented in Geneious R10 and the PhyML webserver (74). For *Diptericin* evolution in Figure S3, sequences were also codon-aligned using MAFFT.

### Fly Stocks and Strain Information

The following strains were used in this study for gene expression analysis and Sanger sequencing: *D. melanogaster* (DrosDel iso *w*^1118^), *D. pseudoobscura, D. virilis, D. immigrans, D. innubila, D. testacea*, and *D. neotestacea*. *Drosophila pseudoobscura* and *D. immigrans* were generously provided by Ben Longdon and correspond to strains used in Duxbury et al. (75). *Drosophila innubila* used in this study is the same as the genome-sequenced strain from Hill et al. (76). *Drosophila virilis* was a gift from Richard Benton corresponding to Sackton et al. (28). Steve Perlman kindly provided Testacea group flies. The *D. testacea* strain used corresponds to the wild-type *D. testacea* described in Keais et al. (77) cleared of *Wolbachia* symbionts by the Perlman lab. The *D. neotestacea* strain is the same as used in Hanson et al. (24). *Drosophila melanogaster, D. pseudoobscura*, and *D. virilis* were reared on standard food medium for *D. melanogaster* and reared at 25°C. *Drosophila immigrans, D. innubila*, and *D. neotestacea* were reared on Nutri Fly^TM^ instant formulation supplemented with a piece of *Agaricus bisporus* mushroom, and reared at 22°C. All species were kept at 22°C during the course of infection. All flies used in this study were previously shown to be negative for *Wolbachia, a* common endosymbiont of *Drosophila*.

### Gene Expression Analysis

Infections, RNA extraction, and cDNA synthesis were performed as previously described (51). Pooled samples of 6 flies (3 males and 3 females) were used for each replicate experiment, and three repeats were performed (18 total flies per treatment per species). Flies were frozen either 6 hpi (Unchallenged, *Escherichia coli (E. coli*)] or 24hpi (*M. luteus, C. albicans*) at −20°C in TRIzol. Quantitative PCR was performed on a LightCycler 480 (Roche) in 96-well plates using Applied Biosystems PowerUP Master Mix. Error bars represent one standard deviation from the mean. Statistical analysis was performed using One-way ANOVA with Holm's-Sidak *post-hoc* comparisons to the treatment that induced expression most in each species for each gene (marked as “ref”); e.g., the *E. coli* treatment was the point of comparison for *Dpt*, and *C. albicans* for Bomanin in *D. melanogaster*. *P*-values are reported as: not significant = ns, <0.1 = ^•^, <0.05 = ^*^ <0.01 = ^**^ and <0.001 = ^***^.

We note the pattern of *Dpt* induction we observed conflicts with a previous report that *Dpt* is not inducible in *D. neotestacea* (24), which is likely explained by measuring alternate *Dpt* isoforms. Primers used in this study, additional expression data for AMPs in different species, and *D. neotestacea Dpt* primer comparisons suggesting alternate *Dpt* isoforms can be found in Supplementary Data File 1.

### Diptericin Evolution in Drosophila

We identified segregating putative null alleles in *Drosophila melanogaster* populations by visually inspecting alignments of re-sequenced individual inbred lines (Figures 3A,B) or pool-seq alignments (North American populations in Figure 3C) (57, 78). We found three classes of putative null alleles: (a) premature stop codons, (b) deletions that disrupt core parts of the transcript (i.e., the start codon), and (c) deletions that are in frame but were associated with reduced immune defense against *P. rettgeri* in prior studies (79). Thus, while the counts in Figure 3A for the USA represent individual inbred lines, those in Figure 3C represent the proportion of reads at a given site carrying the particular null allele.

## Data Availability Statement

The datasets generated for this study can be found in the GenBank database, accession numbers MN311474-MN311476.

## Author Contributions

MH performed live specimen experiments. MH and RU screened and analyzed data. MH, BL, and RU wrote the manuscript.

### Conflict of Interest

The authors declare that the research was conducted in the absence of any commercial or financial relationships that could be construed as a potential conflict of interest.

## Supplementary Material

Figure S1 Alignment of Metchnikowin (*Mtk*) and Mtk-like sequences. We recovered *Mtk* genes from mushroom-feeding flies (brown highlight) that retain the major portion of the *Mtk* mature peptide. We also recovered a clear *Mtk* ortholog in the outgroup Drosophilid *P. variegata* (Pvar\Mtk), which resembles Mtk-like sequence in the Brachyceran fly *Solenopsis brevicornis (S. brevicornis)* (Sbre\Mtk-like). Other Mtk-like sequences from Brachycerans are also shown, and the full open reading frame is shown for all sequences.

Figure S2 The *Drosophila busckii Diptericin* gene region encodes 6 copies of the subgenus Drosophila-restricted *Dpt* (*DptC* clade, see Figure S3) and 3 copies of *DptB*. There are an additional 3 *Diptericin* pseudogenes apparent in the gene region.

Figure S3 Maximum likelihood tree showing that Tephritid *Diptericins* paraphyletically cluster within the *Drosophila DptB* clade, though bootstraps for exact sorting are poor (as expected of paraphyletic clustering); 100 bootstraps, where bootstraps <30 are not shown. Major *Dpt* clades are highlighted as follows: Blue, *DptA*; Red, *DptC*; Green, *DptB*; Teal, Tephritid *Dpts*.

Supplementary Data File 1 **Annotated life histories:** Annotations of feeding ecology in larvae and adults of Diptera in this study. Used in Figure 1. **Datasets used in this study:** Datasets and their accesions and quality scores for diverse Diptera used in this study. Used in Figure 1. Primers used in this study: Primers used in qPCR analysis or Sanger sequencing. qPCR reactions were run with a 60°C annealing and extension phase. Used in Figure 2. **qPCR data from alt. species:** qPCR data from initial explorations using Drosophila pseudoobscura, Drosophila immigrans, and Drosophila testacea (1 Experiment). Remarked as “not shown” in manuscript. **Dneo DptC primer comparison:** Comparison of Dneo\Dpt induction as measured by primers from Hamilton et al. (2014) and universal Testacea group Dpt primers from this study. Revises interpretation of Hamilton et al. (2014) and (24).
