# Supplementary figures and images for: Dynamic Evolution of Antimicrobial Peptides Underscores Trade-Offs Between Immunity and Ecological Fitness
Source: Front Immunol. 2019 Nov 8;10:2620. doi: 10.3389/fimmu.2019.02620 (PMC6857651; doi:10.3389/fimmu.2019.02620)

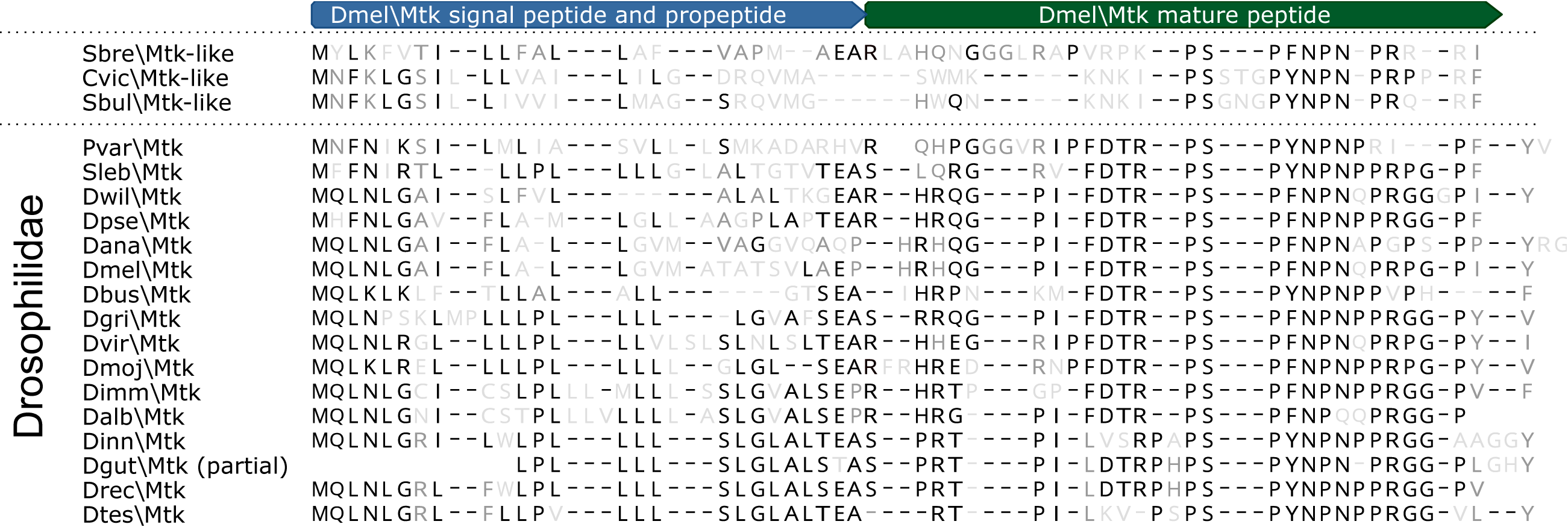

Supplement: Figure S1 — Alignment of Metchnikowin (Mtk) and Mtk-like sequences. We recovered Mtk genes from mushroom-feeding flies (brown highlight) that retain the major portion of the Mtk mature peptide. We also recovered a clear Mtk ortholog in the outgroup Drosophilid P. variegata (Pvar\Mtk), which resembles Mtk-like sequence in the Brachyceran fly Solenopsis brevicornis (S. brevicornis) (Sbre\Mtk-like). Other Mtk-like sequences from Brachycerans are also shown, and the full open reading frame is shown for all sequences. [file Image_1.tif]

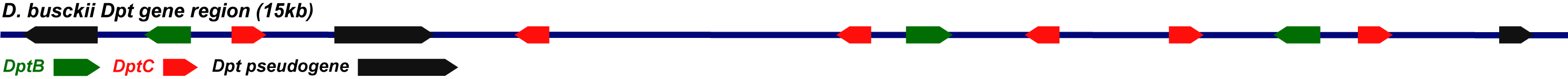

Supplement: Figure S2 — The Drosophila busckii Diptericin gene region encodes 6 copies of the subgenus Drosophila-restricted Dpt (DptC clade, see Figure S3) and 3 copies of DptB. There are an additional 3 Diptericin pseudogenes apparent in the gene region. [file Image_2.tif]

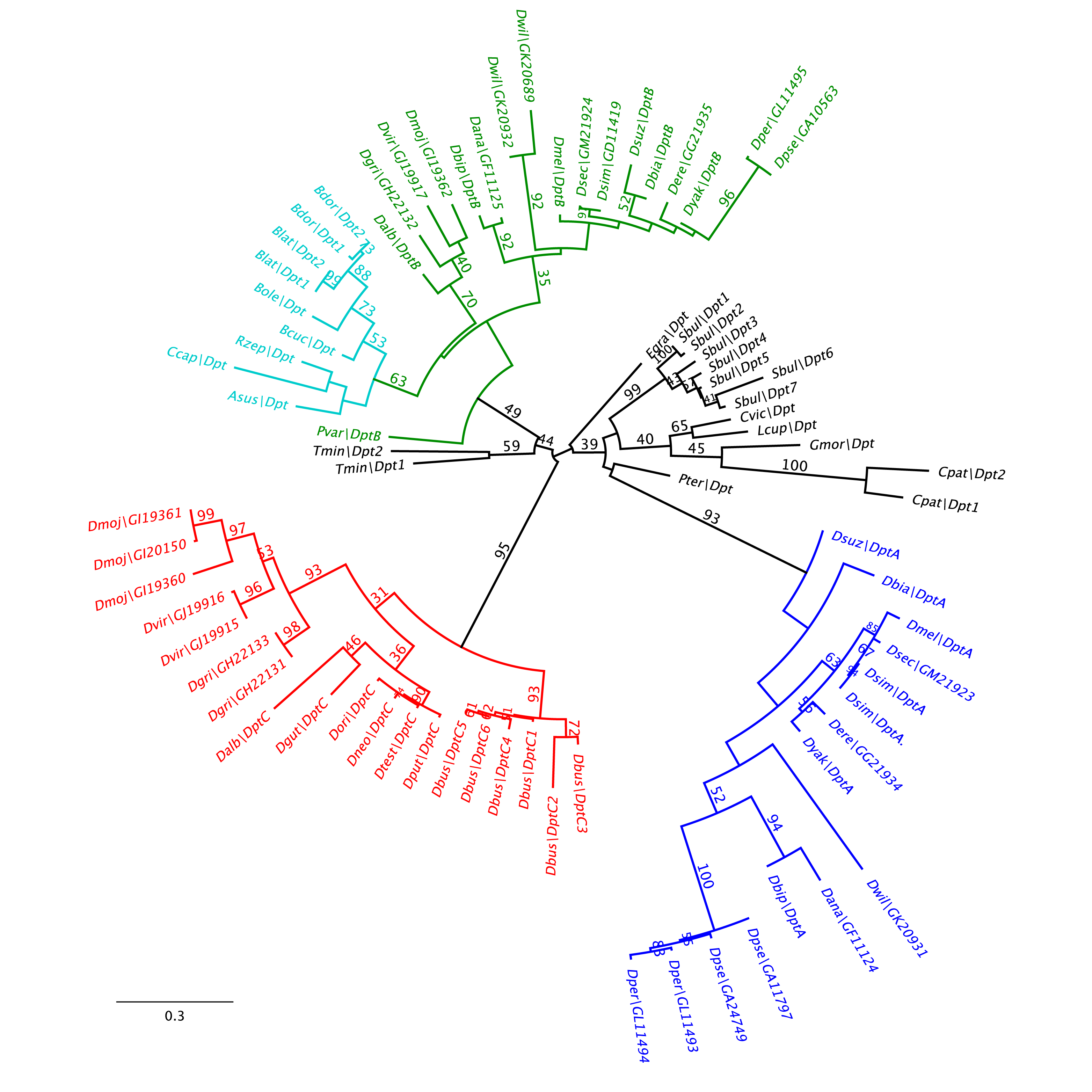

Supplement: Figure S3 — Maximum likelihood tree showing that Tephritid Diptericins paraphyletically cluster within the Drosophila DptB clade, though bootstraps for exact sorting are poor (as expected of paraphyletic clustering); 100 bootstraps, where bootstraps <30 are not shown. Major Dpt clades are highlighted as follows: Blue, DptA; Red, DptC; Green, DptB; Teal, Tephritid Dpts. [file Image_3.TIF]
